# Leveraging primary care data to understand military veteran health in England: feasibility study and matched-control comparison of recorded conditions

**DOI:** 10.1186/s12916-026-05005-5

**Published:** 2026-06-30

**Authors:** Pamela Almeida-Meza, Alexandru Dregan, Bethany Croak, Shehan Hettiaratchy, Nicola T. Fear, Sharon A.M. Stevelink

**Affiliations:** 1 Department of Psychological Medicine, Institute of Psychiatry, Psychology & Neuroscience, King’s College London, London, UK; 2 King’s Centre for Military Health Research, King’s College London, London, UK; 3 Centre for Injury Studies, Imperial College London, London, United Kingdom; 4 Academic Department of Military Mental Health, King’s College London, London, UK

**Keywords:** Military veterans, primary care, electronic health records, Clinical Practice Research Datalink (CPRD)

## Abstract

**Background:**

Evidence on the health needs of UK veterans accessing primary care is limited. Electronic health records (EHRs) offer an opportunity to address this evidence gap if veterans are correctly identified. This study validated the identification of veterans in the Clinical Practice Research Datalink (CPRD) and assessed its feasibility for examining their health compared with non-veterans.

**Methods:**

We conducted a matched cohort study in CPRD, the largest primary care database in the UK, identifying veterans using military-related codes in patients’ EHRs. Each veteran was matched to one or two non-veterans on age, gender, practice, and index date. Validation was undertaken through general practitioner confirmation of veteran status. We compared demographics, risk factors and recorded health conditions using descriptive statistics. Poisson regressions assessed the association between veteran status and various physical and mental health conditions.

**Results:**

122,484 veterans and 244,573 matched non-veterans were identified. 95% were captured using definite military terms, and validation showed substantial agreement with GP records. Veterans had higher recorded prevalence across all conditions. The largest differences were observed for PTSD (adjusted prevalence ratio [aPR] 16.43, 95% CI 14.89–18.13), followed by Alzheimer’s disease (aPR 2.74, 95% CI 2.56–2.92), alcohol use disorder (aPR 2.23, 95% CI 2.09–2.39), hearing loss (aPR 2.09, 95% CI 2.02–2.15), and osteoarthritis (aPR 1.98, 95% CI 1.93–2.03). Recorded prevalence was also higher among veterans for COPD (aPR 1.83, 95% CI 1.77–1.89), depression (aPR 1.83, 95% CI 1.79–1.87), prostate cancer (aPR 1.83, 95% CI 1.73–1.94), coronary heart disease (aPR 1.78, 95% CI 1.72–1.84), lower back pain (aPR 1.73, 95% CI 1.70–1.77), and myocardial infarction (aPR 1.65, 95% CI 1.56–1.75). Differences for anxiety and breast cancer were modest.

**Conclusions:**

This study establishes a foundation for UK veterans’ health research using primary care data. Provided that identification and recording of veteran status improve in NHS records, CPRD offers considerable potential to monitor veteran health trends, identify needs, and evaluate interventions at scale.

**Supplementary Information:**

The online version contains supplementary material available at https://doi.org/10.1186/s12916-026-05005-5.

## Background

In the UK, a veteran is defined as someone who has served (Regular or Reserve) for at least one day in the UK Armed Forces. In England and Wales, ~ 4% of the population, equivalent to 1.8 million people, identify as veterans [1]. Understanding the mental and physical impacts of military service is crucial to ensuring better support for veterans as they transition to civilian life. Military service and deployments can involve prolonged periods of weight-bearing activity and more extreme exposures such as combat, which can have lasting effects on an individual’s mental and physical health. In addition, the transition to civilian life can be a difficult emotional adjustment as individuals recreate a new identity, establish new social networks and are often required to find civilian employment [2]. Such challenges may contribute to psychological distress and poorer mental health outcomes after service.

Despite the importance of this issue, evidence on the health of UK veterans remains limited. Unlike some countries, such as the United States, where integrated administrative systems (e.g. the Department of Veterans Affairs) have facilitated large-scale research on veterans’ health, the UK lacks a comparable national infrastructure for monitoring veterans within healthcare records. Consequently, most existing knowledge stems from survey-based and cohort studies [3, 4]. Although valuable, survey-based studies are often limited by selection bias with some groups such as ethnic minorities less likely to participate [5]. They may also have limited representation of other groups in the Armed Forces, such as women and early service leavers, which hampers our ability to understand the needs of these sub-groups. This is particularly important as such groups may be at greater risk of poor health outcomes and therefore require tailored interventions to address their specific needs [6].

Until the introduction of a military service question in the 2021 Census, it was not possible to reliably estimate the number of veterans in the UK, and the UK Ministry of Defence does not routinely track the long-term outcomes of service personnel after they leave. Instead, veterans access healthcare through the National Health Service (NHS) [7]. When veterans attend GP or hospital appointments, details such as symptoms, diagnoses, test results, and prescriptions are recorded in electronic health records (EHRs), providing a valuable resource for research. However, identifying veterans within these records remains difficult. Although an Armed Forces service flag exists and GP practices are encouraged to ask about military service, this information is inconsistently recorded [8].

In the UK, veteran recording practices vary regionally. In Scotland, when someone joins the Armed Forces, NHS Scotland adds an “Exit to Armed Forces” code to their record, followed by a “Rejoin from Armed Forces” code if they re-register after leaving service. Using this marker in secondary care data, namely NHS hospital admissions and disease specific registers, Bergman and colleagues identified trends in Scottish veterans’ health including an increased risk of smoking-related diseases, musculoskeletal problems, cardiovascular disease compared to civilians and an increased risk of type 2 diabetes amongst veterans with PTSD [9]. Whilst hospital records provide important insight into acute and severe health problems, they only capture a limited portion of the healthcare needs experienced by veterans. To gain a fuller picture, primary care data are crucial, since GPs are the first port of call for most health concerns and capture a wide range of conditions, including chronic illnesses, mental health difficulties, and early-stage conditions that may never result in hospital admission yet have substantial implications for quality of life and long-term wellbeing.

There is currently very limited evidence detailing the conditions veterans present with in primary care settings, particularly to general practitioners (GPs). To our knowledge, studies from the University of Chester’s Westminster Centre for Research in Veterans are the only to have used English primary care data to examine veteran health. Using records from 13 GP practices in the North West of England, researchers analysed data from around 2,700 veterans and 2,700 demographically matched controls. Over one-third had a recorded mental health condition, most commonly depression (15%). Compared to non-veterans, veterans showed higher rates of PTSD, hypertension, type 2 diabetes, and alcohol-related health problems [10–12]. However, these studies are limited in geographical scope and size, rely on cross-sectional extraction of ever-recorded diagnoses, and did not account for potential differences in NHS registration history between veterans and controls, which may influence observed morbidity.

The Clinical Practice Research Datalink (CPRD) is a trusted research service that collects anonymised primary care EHRs from practices across the UK [13]. As the largest primary care database in the UK, it offers significant potential for examining the health conditions with which veterans present to primary care and for addressing gaps in the evidence base, such as the health of ethnic minority and ex-servicewomen [14]. This study, therefore, aimed to:

1. Validate the accuracy of veteran status in CPRD by comparing CPRD identified veterans and non-veterans with confirmation from general practices across England.
2. Explore the utility and feasibility of CPRD as a novel, large scale data asset to identify the healthcare needs of UK veterans and to assess the burden of recorded physical and mental health conditions of veterans compared to non-veterans.

## Methods

### Data

A matched cohort study design was implemented using CPRD Aurum which contains anonymised EHRs from primary care practices in England and Northern Ireland using EMIS software. For this study, we used the September 2024 release of CPRD Aurum [15]. This release included data from approximately 50 million patients (including those no longer registered with participating practices and deceased patients), with around 17 million (24% of the UK population) currently registered with a practice [15]. The database captures key clinical information such as symptoms, diagnoses, prescriptions, tests, and referrals. Previous studies have shown that CPRD data is reliable and accurate for researching a wide range of health conditions [16–19].

### Study population

#### Veteran identification strategy

To identify veterans in CPRD, we classified relevant Systematized Nomenclature of Medicine – Clinical Terms (SNOMED-CT) medical codes [20]. We reviewed common terms reported in the literature and used a list of medical codes to identify veterans in secondary mental healthcare records [21, 22]. We also searched CPRD’s medical dictionaries for the following terms relating to military and veteran status: *military*, *veteran*, *armed forces*, *combat*, *army*, *air force*, *raf*, *navy*, *marine*, *royal engineer* and *corps* where the asterisk represented a wildcard search operator. The terms *reserve*, *deploy, *service person*, *serving*, *soldier* returned no results or resulted in codes already defined using the terms listed first and were therefore excluded from the final search strategy. Medical codes were cross-checked against military and veteran-related terms in the SNOMED-CT browser to assess completeness. A group of veteran health experts (see Acknowledgements section), which included clinicians with experience supporting veterans in the NHS, reviewed the resulting codelist to ensure it accurately reflected veteran status.

A total of 121 codes were included for extraction from CPRD Aurum including terms relating to both regular and reserve Armed Forces service. The codelist used to identify veterans is available on Zenodo https://doi.org/10.5281/zenodo.18880526.

The codes were categorised as definite or probable based on the certainty with which they indicated veteran status (see Additional File 1: Table S1). Codes were considered definite if they explicitly stated veteran status (e.g., “military veteran” or “history of military service”), and probable if they referred to a military role or active duty without confirming that the patient was no longer serving (e.g., “Armed Forces” or “exposed to combat during service”). Patients could have multiple veteran related codes in their EHRs.

All patients identified using the codelist as veterans between 1 January 1987, when data collection for CPRD started [23], to 30 September 2024 were included. Because it is unlikely that younger individuals are veterans, only those aged 18 and older were included in the analysis. For veterans, the “index date” was defined as the date of the first veteran-related medical code recorded in their EHR, marking their initial identification as a veteran within the NHS.

Where available, codes were assigned a category based on branch of service: Royal Navy (including Royal Marines), British Army, or Royal Air Force (RAF) (see Additional File 1: Table S1). Additionally, we identified veterans eligible for National Service era conscription if they were aged 25 years or over in 1963 when the last National Service person was discharged [24].

#### Selection of comparison group

The veteran population differs from the general population on key characteristics such as age and gender [25], with veterans tending to be older and identifying as men, both of which influence health risks and healthcare use. Veterans also have distinct patterns of NHS contact, as healthcare during service is provided by the UK Ministry of Defence, resulting in fewer NHS records before discharge.

To minimise confounding and ensure comparability, we used individual matching on age, gender, primary care practice, and index date. For non-veterans, “index date” was defined as the date of first primary care registration in that practice. This approach reduces the risk that differences between groups reflected demographic structure, practice-level variation, or unequal follow-up time. Each veteran was matched to two non-veterans on these variables where possible. In some cases, only one eligible match was available due to limited numbers in the non-veteran pool, particularly at some variable extremes (e.g., the youngest and oldest age groups). A 2:1 matching ratio was chosen to increase statistical power while preserving comparability between groups.

### Primary care data

#### Demographic characteristics

Demographic characteristics included age (18 and over), gender (men or women), ethnicity (Asian, Black, Mixed, Other, or White), and UK region (based on the location of primary care practice). Key demographic factors (e.g., gender and age) are well recorded in primary care data. However, missing data was identified for ethnicity, which is a key variable to consider when investigating health outcomes. Therefore, linked Hospital Episode Statistics data was used to backfill ethnicity, reducing the overall missing for this variable from 15% to 7%.

#### Follow-up characteristics

Each patient’s medical records were followed up with the ‘start date’ defined as the latest of the registration date, index date, or protocol start date (1 January 1987), and ‘end date’ defined as the earliest of the registration end date, last CPRD collection date for the practice, date of death, or protocol end date (30 September 2024). Additionally, date of death was obtained from CPRD, and primary care consultations were summarised as the average number of consultations per patient per year.

#### Health risk factors and outcomes

Health risk factors included smoking status (never, ex-smoker, and smoker), body mass index (BMI) (underweight: ≤18.49, normal weight: 18.50 to 24.99, overweight: 25 to 29.99, obesity class I: 30 to 34.99, and obesity class II or higher: ≥35), and blood pressure (normal blood pressure: Systolic < 120 and Diastolic < 80, pre-hypertension: Systolic 120–139 or Diastolic 80–89, and hypertension: Systolic ≥ 140 or Diastolic ≥ 90).

The main health outcomes in this study were a set of common physical and mental conditions selected based on previous evidence [3, 9, 11, 26–29] and informed by discussions with our group of veteran health experts. These included alcohol use disorder, Alzheimer’s disease, breast cancer, coronary heart disease (CHD), chronic obstructive pulmonary disease (COPD), hearing loss, lower back pain, myocardial infarction, osteoarthritis, prostate cancer, anxiety, depression, and PTSD. Given that breast and prostate cancer are sex-specific, we examined the distribution of these conditions among men and women in our sample. Breast cancer was observed in both men and women and was therefore analysed in the full sample. On the other hand, prostate cancer analyses were restricted to men because the number of cases among people identifying as women was extremely small (0.04%).

All risk factors and outcomes were defined using lists of medical codes recorded in the patients’ EHRs by GPs, using codelists developed previously, as reported in the literature [16, 17, 30]. For each condition, patients with at least one relevant medical code recorded at any time during their observation period were classified as ‘cases’, while those without the code were classified as ‘non-cases’, ensuring each patient contributed only once per condition. The record closest to the patients’ start date was taken as the date of occurrence.

### Analysis

#### Validation study

To assess the accuracy of identifying veterans in CPRD, a validation study was carried out using CPRD’s Providing Online Verification of EHR (PROVE) service [31]. This service enables researchers to contact general practices to verify information recorded in patients’ EHRs. Agreement between CPRD and GP records was assessed using Cohen’s kappa. Positive predictive value (PPV) and negative predictive value (NPV) were also calculated to quantify the accuracy of the CPRD identification.

Given the time and resources available, our target was to obtain confirmation of veteran status for 100 patients (both veterans and non-veterans). Anticipating incomplete responses from practices, we oversampled by submitting 500 patient identifiers. PROVE subsequently contacted 50 general practices across England, providing each with 10 patient identifiers (five identified in CPRD as veterans and five as non-veterans). Half of the selected practices were accredited as “Veteran Friendly”. The Veteran Friendly Practice accreditation programme is a voluntary initiative for GP practices in England. Accredited practices appoint a clinical lead for veterans and ask patients at registration whether they have served in the UK Armed Forces. This information is then coded into the patients’ EHRs, thus Veteran Friendly practices are more likely to have higher quality data on veteran status [32, 33]. GPs were asked to confirm veteran status for each patient according to the information available in their practice records.

#### Health profile

Descriptive statistics were used to compare the socio-demographic, follow-up, mental and physical health profiles of veterans accessing primary care with a non-veteran comparison group. To assess the burden of recorded diagnoses within the study over the follow-up period, prevalence estimates were calculated as the proportion of individuals with a recorded diagnosis of each condition at any time during the observation period.

Poisson regression models with robust standard errors were used to estimate the prevalence ratios for the association between veteran status and the recorded health outcomes. To address missing data on ethnicity, BMI and smoking status, multiple imputation by chained equations (MICE) was performed [34, 35]. The imputation model included relevant demographic and outcome variables. A total of 20 imputed datasets were generated.

#### Sensitivity analysis

Three sensitivity analyses were carried out. The first two explored alternative assumptions for missing data [34]. First, to assess the impact of missing data, we conducted a complete case analysis. Second, to explore the potential influence of missing not at random in the health risk factors, we recorded missing values for BMI and smoking status to the “healthy” category, under the assumption that these measures may be more likely to be recorded when clinically relevant. For this second sensitivity analysis, missing ethnicity was retained using the missing indicator method. And third, to ensure that the findings are not biased by incomplete or inconsistent case ascertainment, the last sensitivity analysis was conducted restricting the sample to observations from 2014 onwards, when a larger proportion of veterans were identified.

This study was reported in accordance with STROBE guidelines for observational studies (see Additional File 1: Table S2).

All analyses were conducted using Stata version 19 MP (StataCorp LLC).

## Results

### Veteran identification and non-veteran matching

**Fig. 1:**
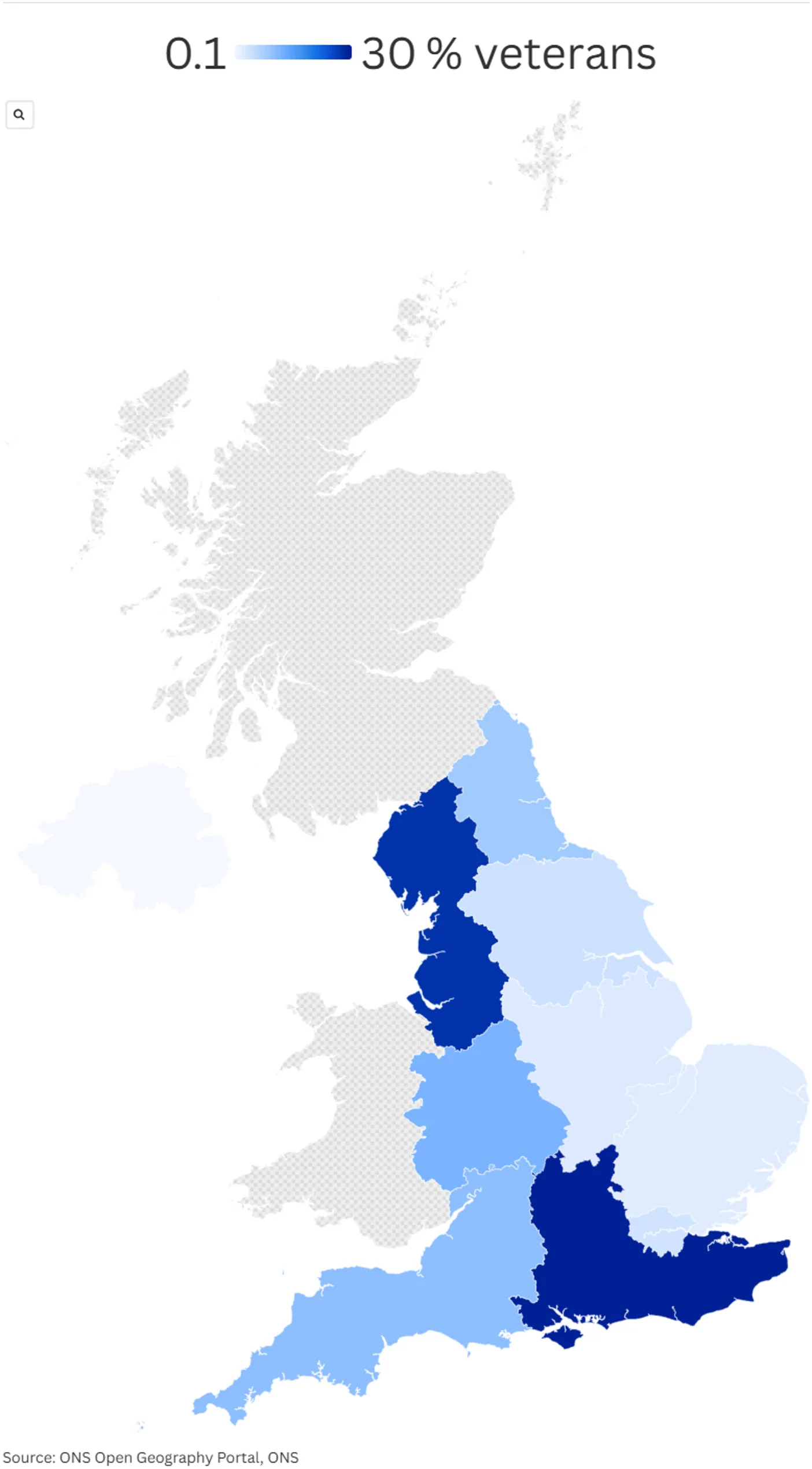
Proportion of veterans across the UK and by English regions, identified in CPRD Aurum

The study sample included 122,484 veterans and 244,573 matched non-veterans, totalling 367,057 individuals. Matching was achieved at 2:1 ratio for 122,089 participants, and 1:1 on 395 participants. From the 122,484 veterans, 116,446 (95%) were identified with definite terms, therefore the majority of the cohort was classified with high certainty codes as veterans. The veteran and non-veteran groups were matched on age, gender, index date, and primary care practice, with as expected, nearly identical distribution across these variables (see Table 1; Fig. 1).

**Table 1:** CPRD Aurum sample (veteran and non-veterans) descriptives (*N* = 367,057)

		Non-veterans 244,573		Veterans 122,484	
		*N*	% or Mean (SD) or Median [IQR]	*N*	% or Mean (SD) or Median [IQR]
Gender*					
	Men	196,472	80.33	98,404	80.34
	Women	48,101	19.67	24,080	19.66
Age at index (years)*			52.55 (20.99)		52.54 (20.99)
	18–25	26,352	10.77	13,200	10.78
	26–35	39,470	16.14	19,772	16.14
	36–45	35,359	14.46	17,713	14.46
	46–55	36,918	15.09	18,492	15.10
	56–65	33,964	13.89	17,008	13.89
	66–75	26,950	11.02	13,494	11.02
	76–85	29,122	11.91	14,579	11.90
	86–95	15,354	6.28	7,684	6.27
	96+	1,084	0.44	542	0.44
**Ethnicity**					
	Asian	11,839	5.33	3,096	2.61
	Black	4,379	1.97	1,853	1.56
	Mixed	3,089	1.39	957	0.81
	Other	19,256	8.68	8,414	7.10
	White	183,371	82.62	104,166	87.91
	*Missing*	*22*,*639*	*9.26*	*3*,*998*	*3.26*
Year of index*					
	1987–1990	1,546	0.63	776	0.63
	1991–2000	9,671	3.95	4,843	3.95
	2001–2010	26,975	11.03	13,499	11.02
	2011–2020	139,872	57.19	70,019	57.17
	2021–2024	66,509	27.19	33,347	27.23
**Follow-up time (years)**†			3.00 [1.16 to 5.73]		2.90 [1.24 to 5.57]
	< 1 year	53,048	21.69	24,288	19.83
	1 to 2 years	69,230	28.31	38,407	31.36
	3 to 4 years	46,358	18.95	23,828	19.45
	5 to 6 years	35,124	14.36	16,523	13.49
	> 7 years	40,813	16.69	19,438	15.87
**Primary care consultations**			6.50 [4.00 to 11.00]		10.11 [6.00 to 16.71]
	1 to 9	171,866	70.82	60,087	49.11
	10 to 19	53,517	22.05	40,872	33.41
	20 to 29	12,584	5.19	14,447	11.81
	≥ 30	4,707	1.94	6,938	5.67
	*Missing*	*1*,*899*	*0.78*	*140*	*0.11*
**Mortality**					
	Deceased	17,175	7.02	12,816	10.46
**Year of death**					
	1990–2000	768	4.47	61	0.48
	2001–2010	3,063	17.83	939	7.33
	2011–2020	8,781	51.13	5,687	44.37
	2021–2024	4,563	26.57	6,129	47.82
**Service branch**					
	Royal Navy (including Royal Marines)	-	-	5,723	14.76
	British Army	-	-	28,015	73.23
	Royal Air Force	-	-	4,170	10.75
	Multiple mentioned	-	-	876	2.26
	*Missing*	-	-	*83*,*700*	*68.34*
**National Service Era**					
	Post-National Service^+^	-	-	103,297	84.34
	Eligible during National Service	-	-	19,187	15.66

Category percentages are based on complete cases; missing percentages are based on the full sampleSD: Standard deviation; IQR: Interquartile Range*Matching variable†Start date calculated as maximum value between registration date, index date, and protocol start date (01 January 1987) and end date calculated as minimum value between registration end date, last CPRD collection date for the practice, date of death, or protocol end date (30 September 2024)^+^Defined as Post-National Service if veterans were under 25 in 1963, when the last National Service person was discharged

### Validation study

From the 500 patient IDs identified in CPRD and sent to PROVE, we received responses for 95 patients. However, patient data could not be accessed for 34 individuals who had left the practice or had died (see Additional File 1: Figure S1 for study flowchart). Most veterans (*n* = 35) were correctly identified (*n* = 24) but 31% (*n* = 11) of CPRD veterans were classified as non-veterans by GPs. However, all ‘false positives’ identified by GPs contained definite terms related to military service in CPRD (e.g., ‘left military service’ and ‘military veteran’), suggesting these are likely genuine veterans. All non-veterans (n = 26) were correctly classified by both sources. Agreement between CPRD and GP records was substantial (Cohen’s κ = 0.65). The PPV (68.6%) indicated moderate concordance for veteran identification, while the NPV (100%) showed that CPRD non-veteran classification aligns with GP records.

### Demographic and administrative characteristics

The demographic and administrative characteristics of veterans and matched non-veterans are presented in Table 1. Most of the sample comprised men (80.34%), and individuals identifying as White (84.47%). The mean age at index was 52.54 (SD = 20.99) years. Most veterans (84.38%) were identified as such in primary care after 2011. Veterans and non-veterans were followed up for approximately three years. Veterans had a higher median number of GP consultations over the follow-up period compared to non-veterans, 10.11 (IQR = 6.00-16.71) vs. 6.50, (IQR = 4.00–11.00), reflecting greater healthcare needs and/or differences in health-seeking behaviours.

Despite matching on age and having comparable follow-up durations, veterans had a higher proportion of deaths compared to non-veterans (10.46% vs. 7.02%). Additionally, veteran deaths were heavily skewed to more recent years while non-veterans had a relatively more even spread of mortality across earlier years.

Only 31.67% of veterans had service branch information; among these, the majority had served in the British Army. Additionally, only 15.66% of the veteran sample would have been eligible to serve during National Service era.

### Comparative health profile

The matched veterans and non-veteran sample differed across some health risk factors (see Table 2). Compared to non-veterans, veterans were less likely to have never smoked (46.36% veterans vs. 53.84% non-veterans), and more likely to be ex-smokers (33.37% vs. 26.12%). Furthermore, the recorded prevalence of hypertension was similar between veterans and non-veterans (~ 33%). For BMI, veterans were less likely than non-veterans to be underweight (2.03% vs. 4.32%), but more likely to be overweight (38.28% vs. 36.31%), or have obesity (class I: 21.31% vs. 18.94%). Importantly, across all health risk factors, veterans had substantially fewer missing data than non-veterans, suggesting more complete health monitoring or recording in this group.

**Table 2:** CPRD Aurum sample (veteran and non-veteran) health risk factors

		Non-veterans 244,573			Veterans 122,484		
		*N*	% (95% CI)*	% (95% CI)†	*N*	% (95% CI)*	% (95% CI)†
**Smoker**							
	Never	53,742	51.70 (51.39 to 52.01)	53.84 (53.44 to 54.19)	44,193	45.61 (45.29 to 45.92)	46.36 (46.07 to 46.66)
	Ex-smoker	28,392	27.31 (27.04 to 27.58)	26.12(25.86 to 26.38)	32,807	33.86 (33.56 to 34.16)	33.37 (33.08 to 33.66)
	Smoker	21,809	20.98 (20.74 to 21.23)	20.04 (19.79 to 20.29)	19,894	20.53 (20.28 to 20.79)	20.26 (20.01 to 20.52)
	*Missing*	*140*,*630*	*57.50*	*-*	*25*,*590*	*20.89*	*-*
**Blood pressure**							
	Normal	22,100	19.94 (19.71 to 20.18)	20.33 (20.11 to 20.55)	16,233	18.07 (17.82 to 18.32)	19.11 (18.83 to 19.40)
	Pre-hypertension	50,775	45.82 (45.53 to 46.11)	46.80 (46.52 to 47.07)	43,315	48.22 (47.89 to 48.54)	48.21 (47.90 to 48.52)
	Hypertension	37,942	34.24 (33.96 to 34.52)	32.87 (32.61 to 33.13)	30,285	33.71 (33.40 to 34.02)	32.67 (32.37 to 32.98)
	*Missing*	*133*,*756*	*54.69*	*-*	*32*,*651*	*26.66*	*-*
**BMI**							
	Underweight	4,404	5.15 (5.00 to 5.30)	4.32 (4.20 to 4.43)	1,224	1.48 (1.40 to 1.56)	2.03 (1.93 to 2.13)
	Normal weight	24,990	29.23 (28.92 to 29.53)	30.84 (30.46 to 31.23)	23,035	27.84 (27.54 to 28.15)	27.89 (27.59 to 28.19)
	Overweight	29,937	35.01 (34.69 to 35.33)	36.31 (36.02 to 36.60)	32,155	38.86 (38.53 to 39.19)	38.28 (38.00 to 38.57)
	Obesity class I	16,881	19.74 (19.48 to 20.01)	18.94 (18.68 to 19.20)	17,789	21.50 (21.22 to 21.78)	21.31 (21.03 to 21.58)
	≥Obesity class II	9,295	10.87 (10.66 to 11.08)	9.59 (9.38 to 9.80)	8,538	10.32 (10.11 to 10.53)	10.49 (10.28 to 10.70)
	*Missing*	*159*,*066*	*65.04*	*-*	*39*,*743*	*32.45*	*-*

Category percentages are based on complete cases; missing percentages are based on the full sample*Proportions calculated for patients with complete data on variables†Pooled proportions obtained using multiple imputed datasetsBlood pressure: normal= Systolic < 120 and Diastolic < 80, pre-hypertension= Systolic 120–139 or Diastolic 80–89, and hypertension= Systolic ≥ 140 or Diastolic ≥ 90Body mass index (BMI): underweight: ≤18.49, normal weight: 18.50 to 24.99, overweight: 25 to 29.99, obesity class I: 30 to 34.99, and obesity class II or higher: ≥35

As presented in Table 3, veterans showed a markedly higher recorded prevalence of several physical and mental health conditions compared to non-veterans. This included musculoskeletal conditions such as osteoarthritis (9.94% vs. 4.67%) and lower back pain (11.72% vs. 6.53%), as well as higher recorded prevalence of hearing loss (6.78% vs. 3.08%), COPD (5.92% vs. 2.87%), and CHD (5.46% vs. 2.79%). Similarly, depression (10.78% vs. 5.71%), PTSD (3.06% vs. 0.18%), anxiety (8.98% vs. 5.86%), and alcohol use disorder (1.60% vs. 0.69%), were more commonly recorded among veterans. Multimorbidity was more common among veterans. While a similar proportion of veterans and non-veterans had two recorded conditions (27.83% vs. 27.55%), veterans were more likely to have three recorded conditions (11.57% vs. 8.57%) or four or more conditions (5.43% vs. 3.28%), but less likely to have only one recorded condition (55.17% vs. 60.61%).

**Table 3:** CPRD Aurum sample (veteran and non-veteran) mental and physical health profile

	Non-veterans 244,573		Veterans 122,484	
	*N*	% (95% CI)	*N*	% (95% CI)
**Alcohol use disorder**	1,687	0.69 (0.66 to 0.72)	1,962	1.60 (1.53 to 1.67)
**Alzheimer’s disease**	1,578	0.65 (0.61 to 0.68)	2,234	1.82 (1.75 to 1.90)
**Anxiety**	14,322	5.86 (5.76 to 5.95)	11,000	8.98 (8.82 to 9.14)
**Breast cancer**	712	0.29 (0.27 to 0.31)	524	0.43 (0.39 to 0.47)
**CHD**	6,831	2.79 (2.73 to 2.86)	6,685	5.46 (5.33 to 5.59)
**COPD**	7,028	2.87 (2.81 to 2.94)	7,247	5.92 (5.79 to 6.05)
**Depression**	13,954	5.71 (5.61 to 5.80)	13,204	10.78 (10.61 to 10.96)
**Hearing loss**	7,532	3.08 (3.01 to 3.15)	8,300	6.78 (6.64 to 6.92)
**Lower back pain**	15,959	6.53 (6.43 to 6.62)	14,361	11.72 (11.55 to 11.91)
**Myocardial infarction**	2,499	1.02 (0.98 to 1.06)	2,262	1.85 (1.77 to 1.92)
**Osteoarthritis**	11,426	4.67 (4.59 to 4.76)	12,178	9.94 (9.78 to 10.11)
**Prostate cancer** ^*^	2,347	1.19 (1.15 to 1.24)	2,353	2.39 (2.30 to 2.49)
**PTSD**	439	0.18 (0.16 to 0.20)	3,754	3.06 (2.97 to 3.16)
**Multimorbidity**				
1 condition	33,608	60.61 (60.20 to 61.01)	28,063	55.17 (54.74 to 55.61)
2 conditions	15,277	27.55 (27.18 to 27.92)	14,154	27.83 (27.44 to 28.22)
3 conditions	4,752	8.57 (8.34 to 8.81)	5,885	11.57 (10.30 to 11.85)
4 + conditions	1,817	3.28 (3.13 to 3.43)	2,760	5.43 (5.23 to 5.63)

*Men patients only (*n* = 294,876)CHD: Coronary Heart Disease; COPD: Chronic obstructive pulmonary disease; PTSD: Post-traumatic stress disorder

The results from the regression analyses (Table 4) showed that veteran status was consistently associated with increased recorded prevalence for all physical and mental health conditions examined, although adjustment for ethnicity and further adjustment for BMI and smoking attenuated most associations. In the fully adjusted models, the association with PTSD remained substantially higher than for other conditions, with veterans showing more than a 16-fold higher recorded prevalence (aPR = 16.43, 95% CI 14.89 to 18.13). Similarly, elevated prevalence of Alzheimer’s disease (aPR = 2.74, 95% CI 2.56 to 2.92), alcohol use disorder (aPR 2.23, 95% CI 2.09–2.39), hearing loss (aPR 2.09, 95% CI 2.02–2.15), osteoarthritis (aPR 1.98, 95% CI 1.93–2.03) persisted after adjustment.

**Table 4:** Poisson regression for the association between veteran status and physical and mental health conditions (*N* = 367,057)

	Model 1			Model 2			Model 3		
	PR	95% CI		aPR	95% CI		aPR	95% CI	
**Alcohol use disorder**	2.32	2.17	2.47	2.26	2.12	2.41	2.23	2.09	2.39
**Alzheimer’s disease**	2.82	2.65	3.01	2.71	2.55	2.90	2.74	2.56	2.92
**Anxiety**	1.53	1.49	1.57	1.50	1.47	1.54	1.51	1.47	1.54
**Breast cancer**	1.46	1.31	1.64	1.45	1.30	1.63	1.45	1.29	1.63
**Coronary heart disease**	1.95	1.89	2.01	1.90	1.84	1.97	1.78	1.72	1.84
**Chronic obstructive pulmonary disease**	2.05	1.99	2.12	1.99	1.93	2.05	1.83	1.77	1.89
**Depression**	1.88	1.84	1.93	1.85	1.81	1.89	1.83	1.79	1.87
**Hearing loss**	2.20	2.13	2.26	2.14	2.07	2.20	2.09	2.02	2.15
**Lower back pain**	1.79	1.75	1.83	1.79	1.75	1.82	1.73	1.70	1.77
**Myocardial infarction**	1.80	1.70	1.91	1.75	1.66	1.86	1.65	1.56	1.75
**Osteoarthritis**	2.12	2.07	2.18	2.08	2.03	2.13	1.98	1.93	2.03
**Prostate cancer** ^*^	2.00	1.89	2.11	1.93	1.82	2.04	1.83	1.73	1.94
**Post-traumatic stress disorder**	17.07	15.47	18.84	16.84	15.26	18.59	16.43	14.89	18.13

*Men patients only (*n* = 294,876)PR: Prevalence Ratios; aPR: Adjusted Prevalence Ratios; CI: Confidence IntervalModel 1: Unadjusted; Model 2: Model 1 + ethnicity; Model 3: Model 2 + BMI and smoking

### Sensitivity analysis

First, we repeated the main analyses using complete cases (*n* = 118,674; Additional File 1: Tables S3 and S4). Compared with the multiple imputation analyses, complete-case results underestimated the associations between veteran status and the health outcomes. This attenuation is consistent with non-random missingness in primary care data, where lifestyle factors such as BMI and smoking are more likely to be recorded among individuals with greater healthcare engagement or clinical need [34, 36, 37]. As a result, the complete case analysis may overrepresent patients with poorer health profiles in both veteran and non-veteran groups, thereby narrowing group differences and biasing associations toward the null. This interpretation is supported by the higher number of primary care consultations among those retained in the complete case sample compared with the full matched cohort (veterans: median 11.65 complete case vs. 10.11 full matched cohort; controls: median 10.32 vs. 6.50). In contrast, multiple imputation allows inclusion of the full matched dataset and adjusts for systematic patterns of missingness related to demographic and clinical characteristics, resulting in more reliable and internally consistent estimates.

Second, when missing values for BMI and smoking status were recorded to the “healthy” category (*N* = 367,057; Additional File 1: Table S5), the associations between veteran status and health outcomes were slightly attenuated compared with the multiple imputation analyses but remained consistently elevated across most conditions, supporting the robustness of the main findings.

Third, to assess the potential impact of older or less complete EHRs, we restricted the sample to individuals with an index date in 2014 onwards (*n* = 286,359). Results were consistent with, and in some cases slightly stronger than, the main findings (see Additional File 1: Tables S6 to S9), suggesting that the risk of bias from older data (incomplete records or inconsistent data due to changes in EHR systems or GP recording practices) is minimised.

## Discussion

Using CPRD Aurum, we identified 122,484 veterans and 244,573 matched non-veterans. This study is the first to identify UK Armed Forces veterans within CPRD, demonstrating that routinely collected primary care data can be used to characterise this population at a national scale. As previously reported in the literature [8], the study also highlighted challenges in the identification and recording of veteran status within primary care that need to be addressed to fulfil the potential of CPRD for monitoring veteran health and evaluating interventions.

Our study assessed the reliability of GP records in identifying veterans by asking practices across England to confirm patients’ veteran status. There was substantial agreement between GP information and CPRD data, supporting the validity of veteran status coding while also highlighting challenges in incomplete or inconsistent recording. Furthermore, this study demonstrated that it was feasible to use CPRD to build a health profile of veterans and a comparison group. This allows researchers to harness a nationally representative, routinely collected dataset to characterise both the physical and mental health needs of UK veterans and to compare them directly with the wider population.

### Results in relation to previous literature

Consistent with the demographic profile of veterans reported in the 2021 Census and the 2022 Veteran Survey by the Office for National Statistics [25, 38], the majority of CPRD veterans were men. Women were slightly overrepresented among CPRD veterans (20% vs. 14% in the 2021 Census), which might reflect women’s higher consultation rates in primary care and therefore more opportunities for their veteran status to be recorded in their EHRs [39].

In terms of age distribution, 30% of veterans in the 2021 Census were aged 80 years and older (reflecting the National Service cohort) [25], compared with 14% of CPRD veterans, indicating a younger skew in this dataset. This might partly reflect underreporting of veteran status among older individuals who, having served many decades ago, may not report their status to their GP. Supporting this, only 16% of the CPRD veteran sample were eligible for National Service era conscription. However, individuals who did not serve during this period were often exempt due to medical or occupational reasons, meaning the non-veteran comparison group in older age groups may be under-representative and systematically different [40]. As a result, comparisons in this subgroup can be challenging to interpret and should be treated with caution. Future research using CPRD should consider these limitations when examining older cohorts and, where possible, stratify analyses by service era while accounting for potential selection biases in the non-veteran group.

In line with national trends, CPRD veterans were less ethnically diverse than non-veterans, with most identifying as White, similar to the Census [25]. Regionally, CPRD veterans were overrepresented in the North West (27% vs. 13%) and South East (29% vs. 18%) – aligning with CPRD Aurum population coverage [41], and underrepresented in other regions. The North East (8% vs. 6%) and London (4% vs. 6%) were more closely aligned. Veterans also showed higher overall mortality than non-veterans, particularly after 2021. This contrasts with previous UK findings of lower mortality among veterans [42], but aligns with US evidence suggesting a recent erosion of this healthy soldier effect [42–44]. This recent higher mortality may reflect a combination of cohort effects, delayed consequences of service, or the impact of the COVID-19 pandemic on healthcare access and mortality. Differences may also reflect heterogeneity in ageing and underlying socioeconomic factors, with some individuals, particularly those from more deprived backgrounds, experiencing earlier mortality. Further work is needed to determine whether this reflects a true erosion of the healthy soldier effect in the UK, pandemic impacts, or methodological artefacts related to how primary care data are collected or recorded. Future research should consider linkage to external data sources, such as ONS mortality records, Census data, and where available, Ministry of Defence administrative data on service leavers, to improve ascertainment and interpretation of mortality patterns.

Follow-up duration was similar between veterans and non-veterans (approximately three years), supporting comparability between groups. While this is a relatively short follow-up, it is consistent with the characteristics of CPRD Aurum data, which has a reported median follow-up of four years [41], reflecting the dynamic nature of primary care registration and data capture in this setting.

Our findings generally align with evidence from a recent study by Finnegan and colleagues (2024 and 2025), which used primary care records and compared veteran health outcomes with those of non-veterans in North-West England. This regional study of 5,458 patients (2,729 veterans matched on age and gender to non-veterans) reported that veterans show a higher prevalence of PTSD (2.9% vs. 0.8%), hypertension (20.9% vs. 17.6%), anxiety (12.8% vs. 11.9%) and alcohol dependence (2.5% vs. 1.4%), but lower prevalence of depression (14.9 vs. 17.1%) and dementia (2.1% vs. 2.5%) [11, 12]. In contrast, drawing on a much larger and nationally representative CPRD cohort, we found higher recorded prevalence of depression, anxiety, PTSD, dementia, and AUD among veterans compared with non-veterans. These differences may reflect variation in sample size, geographic coverage, cohort composition, as well as the absence of matching on veteran identification date and the lack of a defined observation period in the Finnegan study.

Furthermore, prevalence was defined as the proportion of individuals with a recorded diagnosis of each condition at any point during follow-up, not as population-level estimates. As such, differences between veterans and non-veterans may reflect not only variations in underlying disease burden, but also differences in healthcare utilisation or recording practices. For example, veterans may be more likely to consult primary care, particularly in the early years after leaving service, leading to higher recorded prevalence for some conditions. However, this effect might be more pronounced for conditions more likely to be managed in primary care (e.g., musculoskeletal conditions) and less so for more serious conditions.

Although previous evidence has suggested that the prevalence of PTSD varies widely in the veteran population depending on study design, sampling, and measurement, with high estimates reported in selected clinical populations [42], our primary care findings indicate a much lower recorded prevalence of 3%. However, this estimate is comparable to other primary care estimates of 2.9% and 3.4% [10, 12] and secondary care estimates of 1.1% [9] and 2.8% [45] suggesting consistency in recorded prevalence across routine data sources. Furthermore, prevalence estimates derived from CPRD often differ from survey-based estimates, particularly for mental health conditions such as PTSD, which are underdiagnosed or under-recorded in primary care [46]. EHRs only capture recognised cases and are influenced by help-seeking, diagnostic, and recording practices, likely leading to underestimation of prevalence estimates. Future research linking CPRD to mental health service datasets could provide a more complete estimate of PTSD among UK veterans.

Our findings are also consistent with evidence from Scottish veteran cohorts, which have shown that veterans are more likely than non-veterans to develop cardiovascular disease, potentially at an earlier age, with smoking identified as the most significant risk factor alongside obesity, inactivity, and poor diet [9]. In our CPRD analysis, veterans had higher recorded prevalence of cardiovascular disease, including coronary heart disease and myocardial infarction, as well as increased prevalence of COPD. We additionally observed higher rates of ex-smoking among veterans, indicating historical exposure to tobacco, with cessation possibly occurring later or following the onset of disease. Such patterns are consistent with smoking contributing to later-life cardiovascular and respiratory disease risk, even after cessation [47].

### Implications for future research, policy, and practice

The ability to identify veterans in CPRD has considerable translational potential for both the healthcare community (NHS and third sector) and the Ministry of Defence, though this potential remains contingent on resolving some of the limitations identified in this study. Although a veteran status code (“military veteran”) is available and recommended by the Royal College of General Practitioners [48], recording remains incomplete and inconsistent across practices [8]. Policymakers and NHS organisations should prioritise the consistent recording of the recommended code within primary care records and practices should be actively supported and incentivised to apply it at registration and during routine consultations. Without such improvements, the utility of CPRD and other routine datasets for veteran health research will remain constrained. Researchers working with CPRD veteran data should also prioritise methodological development, including refining coding strategies, assessing the impact of misclassification and missing data on different study designs, and evaluating the reproducibility of findings across different CPRD databases and time periods.

The size and depth of the CPRD veteran cohort provide an opportunity to refine and prioritise future research questions around veteran health. The findings highlight areas of elevated risk, including musculoskeletal, respiratory, cardiovascular, and mental health conditions. Rather than focusing narrowly on differences in individual conditions, CPRD enables exploration of broader themes such as inequalities in healthcare access and utilisation, participation in screening and interventions programmes, and the clinical and service determinants of health inequalities within veteran populations. Prioritising questions that leverage the strengths of CPRD, such as its longitudinal structure, rich primary care data linked to secondary care and disease registries datasets, and matched non-veteran comparison group, will allow researchers to investigate service needs over time, examine long-term consequences of military service, and inform targeted policy interventions.

Our analysis indicates that veterans experience higher recorded prevalence of hearing loss compared to non-veterans. While further research is required, these findings highlight the potential value of routine monitoring to identify problems early and continue supporting preventative policy during service. Hearing problems are not only a clinical concern but also affect quality of life, being linked to greater loneliness, social isolation, and mental health problems [49, 50]. It also carries economic implications: service-related hearing loss was the most frequently claimed condition against the Ministry of Defence in 2022/2023 [51]. Such analyses can enable early interventions such as the timely use of hearing aids. These interventions could yield significant health benefits, with evidence showing that hearing aids may reduce cognitive decline by nearly 20% [52]. This is one example of the broader policy implications of using CPRD to identify existing and emerging health issues in veterans and draw attention to issues of particular concern, enabling timely and preventative interventions.

Extending analyses beyond England offers an important opportunity for comparative investigations across the UK, given differences in health system policies and programmes. While this study used CPRD Aurum, which is restricted mainly to England, CPRD Gold includes data from Scotland, Wales, and Northern Ireland [23]. Such analyses could identify whether the provision of different health services across the four nations may carry any explanatory power with regard to variability in health status for military veterans.

Notably, veterans were significantly more likely to experience multimorbidity, with a higher proportion having three or more recorded long-term conditions compared to non-veterans. However, this measure of multimorbidity does not capture the specific combinations or underlying relationships between conditions. Thus, the observed patterns may reflect a mixture of shared risk factors, causal relationships between conditions, and differences in healthcare use. CPRD provides a valuable platform for tracking the onset, clustering, and progression of multimorbidity among veterans over time, and for exploring its unique drivers and consequences for healthcare utilisation, prescribing patterns, and mortality. Understanding how multimorbidity develops in this population, including the specific clusters of conditions that arise, will be critical for designing models of care that address the complex and intersecting needs of those who have served.

### Strengths and limitations

A key strength of this study is the robust approach to identifying veterans in primary care records. Despite inconsistent recording of veteran status, the use of carefully reviewed military-related medical codes and terms enabled accurate classification, with 95% of CPRD veterans identified through definite terms. This also minimised the risk of misclassifying non-veterans as veterans. The large, nationally representative CPRD cohort further strengthens the study, allowing comparison of veterans to non-veterans across a wide range of clinically verified health outcomes. In addition, the study benefitted from close involvement of veteran health experts from multiple disciplines, ensuring accurate interpretation of medical coding and enhancing the clinical validity of the findings.

However, the lack of consistent recording of veteran status created challenges for the validation study: in about one-third of cases where CPRD identified someone as a veteran, the GP reported otherwise. However, all these “false positives” had definite military-related terms in CPRD, suggesting they were likely true veterans. It is possible that this discrepancy occurred because practice staff may not have had sufficient time to comprehensively review extensive historical patient records when completing the validation exercise. This interpretation was discussed with the project advisory board (see Acknowledgements section), who considered this the most likely explanation for many of the discordant cases. While CPRD enables identification of veterans with a high degree of accuracy, under-recognition of veterans in primary care and the use of multiple historical coding terms introduce uncertainty and may result in false negatives. Such misclassification would be expected to bias estimates towards the null, meaning that observed differences may be underestimated. This underscores the need for consistent, systematic recording of veteran status in NHS data systems to support both clinical care and research.

The overall extent of missingness is a limitation which can affect the accuracy and interpretability of some measures, since missing data in primary care records may not satisfy the missing at random assumption. To address this, MICE was carried out to account for missing data in covariates, following recommended best practice for handling missing data in UK primary care research [34, 35]. While MICE cannot fully account for data that are missing not at random, its use allows efficient use of the available information and reduces bias compared with complete-case analyses. Notably, sensitivity analyses demonstrated that the magnitude of associations varied depending on how missing data were handled, with more conservative assumptions leading to attenuated effect estimates, although the overall pattern of findings remained consistent. Future work could explore alternative approaches designed for missing not at random mechanisms to assess the robustness of the findings.

An additional consideration is the lower level of missing data observed among veterans compared with non-veterans. On discharge, service personnel are provided with a summary medical record (FMed133), however, this information may not always be recorded in primary care records [53]. Therefore, it is possible that the subset of veterans identifiable within CPRD represents individuals whose service has been explicitly recorded and whose clinical information has been more completely transferred at registration. As such, veterans included in this study may have more complete recording of risk factors than both non-veterans and veterans not captured in EHRs, adding complexity to the interpretation of differences in missingness between groups.

## Conclusions

This study is the first to identify UK Armed Forces veterans within CPRD and assess the feasibility of generating a preliminary national health profile of this population, establishing a longitudinal resource for understanding the health needs of this population. By confirming veteran status against GP records, we demonstrated that routinely collected primary care data can be used to identify veteran status with a good degree of confidence, providing an important proof of concept for building health profiles at scale.

The use of CPRD represents an important first step in advancing the study of UK veterans’ health, offering a nationally representative, real-world dataset that avoids many of the limitations of volunteer-based cohort studies, while also introducing challenges inherent to routinely collected data that require careful investigation and recognition. It enables exploration not only of specific conditions but also of broader patterns of healthcare use, multimorbidity, and health inequalities, with potential for extension across all four nations of the UK through CPRD Gold.

While this study was primarily designed to test feasibility, the results lay strong foundations for future work and point to important areas for future development, including refining approaches to veteran identification, handling missing data, and using advanced analytic approaches alongside traditional statistical methods. As these foundations are strengthened, CPRD has considerable potential to inform both healthcare provision and veteran-specific policy, ensuring that interventions are timely and responsive to the diverse needs of those who have served.

## Supplementary Information

Below is the link to the electronic supplementary material.

Supplementary Material 1: Additional File 1. Tables S1-S9 and Figure S1. Table S1 List of veteran terms used to identify veterans in CPRD Aurum. Table S2. STROBE checklist for reporting observational studies. Figure S1. Flowchart of CPRD data sent to PROVE and GP data received. Table S3. CPRD Aurum variables descriptives for sample with complete data. Table S4. Poisson regressions for the association between veteran status and health conditions using complete case sample. Table S5. Poisson regression for the association between veteran status and health conditions setting missing in health risk factors to “healthy” categories. Table S6. CPRD Aurum sample descriptives for patients identified/registered in 2014 or after. Table S7. CPRD Aurum sample health risk factors for patients identified/registered in 2014 or after. Table S8. CPRD Aurum sample health profile for patients identified/registered in 2014 or after. Table S9. Poisson regression for the association between veteran status and health conditions for patients identified/registered in 2014 or after.

## Data Availability

The data that support the findings of this study are available from the Clinical Practice Research Datalink (CPRD), hosted by the UK Medicines and Healthcare products Regulatory Agency (MHRA). These data are not publicly available due to patient confidentiality and licensing restrictions. Access is granted subject to data access agreements and applicable fees. Further details on the application process are available via the CPRD website (https://www.cprd.com/). The final list of codes and associated terms used to identify veterans in CPRD Aurum available in supplementary material and online in repository (https://doi.org/10.5281/zenodo.18880526).

## Abbreviations

aPR: Adjusted Prevalence Ratio
BMI: Body Mass Index
CHD: Coronary Heart Disease
CI: Confidence Interval
COPD: Chronic Obstructive Pulmonary Disease
CPRD: Clinical Practice Research Datalink
PROVE: Providing Online Verification of EHR
EHR: Electronic Health Record
FiMT: Forces in Mind Trust
GP: General Practitioner
IQR: Interquartile Range
MICE: Multiple Imputation by Chained Equations
NHS: National Health Service
PR: Prevalence Ratio
PTSD: Post Traumatic Stress Disorder
RAF: Royal Air Force
SD: Standard Deviation
SNOMED CT: Systematized Nomenclature of Medicine – Clinical Terms
