# Supplementary material for: Leveraging primary care data to understand military veteran health in England: feasibility study and matched-control comparison of recorded conditions
Source: BMC Med. 2026 Jun 30;24:502. doi: 10.1186/s12916-026-05005-5 (PMC13587321; doi:10.1186/s12916-026-05005-5)
Supplement: Supplementary file 1 [file 12916_2026_5005_MOESM1_ESM.docx]

**Additional File 1**

**Leveraging primary care data to understand military veteran health in England: Feasibility study and matched-control comparative health profile**

Pamela Almeida-Meza, Alexandru Dregan, Bethany Croak, Shehan Hettiaratchy, Nicola T. Fear, Sharon A.M. Stevelink

| **Table S1.** List of veteran terms used to identify veterans in CPRD Aurum | | | |
| --- | --- | --- | --- |
|  | **Term** | **Service branch** | **Definitive term** |
| 1 | Active duty military |  |  |
| 2 | Active serving member of the Army | Army |  |
| 3 | Active serving member of the Army - temporary registration | Army |  |
| 4 | Active serving member of the Army Reserve | Army |  |
| 5 | Active serving member of the Royal Air Force | RAF |  |
| 6 | Active serving member of the Royal Air Force - temporary registration | RAF |  |
| 7 | Active serving member of the Royal Air Force Reserve | RAF |  |
| 8 | Active serving member of the Royal Marines | Royal Marines |  |
| 9 | Active serving member of the Royal Marines - temporary registration | Royal Marines |  |
| 10 | Active serving member of the Royal Marines Reserve | Royal Marines |  |
| 11 | Active serving member of the Royal Navy | Navy |  |
| 12 | Active serving member of the Royal Navy - temporary registration | Navy |  |
| 13 | Active serving member of the Royal Navy Reserve | Navy |  |
| 14 | Acute posttraumatic stress disorder following military combat |  |  |
| 15 | Adjutant General's Corps personnel | Army |  |
| 16 | Administrative officer - RAF | RAF |  |
| 17 | AIR FORCE | RAF |  |
| 18 | Aircraft ejection - military | RAF |  |
| 19 | Airman, aircrew - RAF | RAF |  |
| 20 | Armed forces |  |  |
| 21 | Armed forces reservist |  |  |
| 22 | Armed forces: non-comissioned |  |  |
| 23 | Armed forces: non-commissioned |  |  |
| 24 | ARMY | Army |  |
| 25 | Army - non-commissioned personnel | Army |  |
| 26 | Army officer | Army |  |
| 27 | Army personnel | Army |  |
| 28 | ARMY TRG | Army |  |
| 29 | Army veteran | Army | ✓ |
| 30 | Chronic post-traumatic stress disorder following military combat |  |  |
| 31 | Commissioned officer - Royal Navy | Navy |  |
| 32 | Commissioned officer medical - Royal Navy | Navy |  |
| 33 | Delayed posttraumatic stress disorder following military combat |  |  |
| 34 | Deserted from military service |  | ✓ |
| 35 | Design draughtsman - Royal Engineers | Army |  |
| 36 | Dog trainer - Royal Army Veterinary Corps | Army |  |
| 37 | Duration of military service |  |  |
| 38 | Electrical and mechanical draughtsman - Royal Engineers | Army |  |
| 39 | Engineer - Royal Navy | Navy |  |
| 40 | Exposed to combat during military service |  |  |
| 41 | Finance and systems administrator - Adjutant General's Corps* | Army |  |
| 42 | General mechanic - RAF | RAF |  |
| 43 | Heating, ventilation and air conditioning fitter - Royal Engineers | Army |  |
| 44 | History of serving in armed forces |  | ✓ |
| 45 | History relating to Army service | Army | ✓ |
| 46 | History relating to military service |  | ✓ |
| 47 | History relating to Royal Air Force service | RAF | ✓ |
| 48 | History relating to Royal Navy service | Navy | ✓ |
| 49 | Horse trainer - Royal Army Veterinary Corps | Army |  |
| 50 | Infantry soldier | Army |  |
| 51 | Injury from military training/exercise |  |  |
| 52 | Joined military forces |  |  |
| 53 | Left military service* |  | ✓ |
| 54 | Marine engineer - Royal Navy | Navy |  |
| 55 | Marine engineer (non-commissioned) - Royal Navy | Navy |  |
| 56 | Marine engineer officer | Royal Marines |  |
| 57 | Medically discharged from military service |  | ✓ |
| 58 | Merchant navy medical |  |  |
| 59 | Military administrative status |  |  |
| 60 | Military clerk |  |  |
| 61 | Military services member |  |  |
| 62 | Military services member of ground forces |  |  |
| 63 | Military veteran |  | ✓ |
| 64 | Movement controller - Royal Logistics Corps | Army |  |
| 65 | Musician - Royal Marines | Royal Marines |  |
| 66 | Nurse - RAF | RAF |  |
| 67 | Officer - armed forces |  |  |
| 68 | Officer, armed forces NOS |  |  |
| 69 | Personnel administrator - RAF | RAF |  |
| 70 | Pharmacy technician - RAF | RAF |  |
| 71 | Pharmacy technician - Royal Army Medical Corps | Army |  |
| 72 | Plumber and pipe fitter - Royal Engineers | Army |  |
| 73 | Postal and courier operator - Royal Logistics Corps | Army |  |
| 74 | Princess Mary's Royal Air Force Nursing Service | RAF |  |
| 75 | Regimental accountant - Adjutant General's Corps | Army |  |
| 76 | Regimental administration officer finance - Adjutant General's Corps | Army |  |
| 77 | Regt/corps/command |  |  |
| 78 | Royal Air Force administrative officer, education | RAF |  |
| 79 | Royal Air Force administrative officer, secretarial | RAF |  |
| 80 | Royal Air Force communications systems voice analyst | RAF |  |
| 81 | Royal Air Force data analyst | RAF |  |
| 82 | Royal Air Force electrical engineering technician, telecommunications | RAF |  |
| 83 | Royal Air Force mechanical transport driver | RAF |  |
| 84 | Royal Air Force musician | RAF |  |
| 85 | Royal Air Force navigator, general duties | RAF |  |
| 86 | Royal Air Force operations support manager | RAF |  |
| 87 | Royal Air Force personnel administrator, automatic data processing | RAF |  |
| 88 | Royal Air Force statistical analyst, automatic data processing | RAF |  |
| 89 | Royal Air Force student dental technician | RAF |  |
| 90 | Royal Air Force student laboratory technician | RAF |  |
| 91 | Royal Air Force student pharmacy technician | RAF |  |
| 92 | Royal Air Force student radiographer | RAF |  |
| 93 | Royal Air Force telecommunications operator | RAF |  |
| 94 | Royal Air Force veteran | RAF | ✓ |
| 95 | Royal Army Dental Corps personnel | Army |  |
| 96 | Royal Army Medical Corps personnel* | Army |  |
| 97 | Royal engineers diver | Army |  |
| 98 | Royal Logistics Corps personnel | Army |  |
| 99 | Royal Marines - non-commissioned personnel | Royal Marines |  |
| 100 | Royal Marines veteran | Navy | ✓ |
| 101 | Royal navy limitations |  |  |
| 102 | Royal Navy officer | Navy |  |
| 103 | Royal Navy personnel | Navy |  |
| 104 | Royal navy templates |  |  |
| 105 | Royal Navy veteran | Navy | ✓ |
| 106 | Seaman - Royal Navy | Navy |  |
| 107 | Served in armed forces |  | ✓ |
| 108 | Served in armed forces in combat* |  | ✓ |
| 109 | Served in military service |  | ✓ |
| 110 | Serving in military service |  |  |
| 111 | Signposting to Veterans' Gateway |  |  |
| 112 | Soldier - home service force | Army |  |
| 113 | Special Air Services soldier | Army |  |
| 114 | Supply controller - Royal Logistics Corps | Army |  |
| 115 | Topographic technician - Royal Engineers | Army |  |
| 116 | Trainee - armed forces |  |  |
| 117 | Veterinary technician - Royal Army Veterinary Corps | Army |  |
| *Duplicated terms | | | |

| **Table S2.** STROBE checklist for reporting observational studies | | | |
| --- | --- | --- | --- |
| **Item** | **Recommendation** | **Reported** | **Manuscript location** |
| 1a | Indicate the study design in the title or abstract | Yes | Abstract: Methods mentions 'matched cohort study' |
| 1b | Provide informative and balanced abstract | Yes | Structured abstract (Background, Methods, Results, Conclusions) |
| 2 | Explain scientific background and rationale | Yes | Introduction: Paragraphs describing evidence gap in UK veteran health research |
| 3 | State specific objectives or hypotheses | Yes | Introduction: Study aims |
| 4 | Present key elements of study design early | Yes | Methods: Data section |
| 5 | Describe setting, locations, and relevant dates | Yes | Methods: Data (CPRD Aurum, England/Northern Ireland, 1987–2024) |
| 6a | Eligibility criteria and participant selection | Yes | Methods: Study population, veteran identification strategy |
| 6b | Matching criteria and number of exposed/unexposed | Yes | Methods: Selection of comparison group (2:1 matching) |
| 7 | Define outcomes, exposures, confounders | Yes | Methods: Health risk factors and diagnoses |
| 8 | Sources of data and measurement methods | Yes | Methods: Data and primary care data sections |
| 9 | Describe efforts to address bias | Yes | Methods: Matching strategy, validation study; covariate adjustment, discussion limitations |
| 10 | Explain how study size was arrived at | Yes | Methods: Study population, veteran identification strategy and matching |
| 11 | Explain handling of quantitative variables | Yes | Methods: BMI and blood pressure categories |
| 12a | Describe statistical methods and confounding control | Yes | Methods: Matching and covariate adjustment in Poisson regression |
| 12b | Methods for subgroups or interactions | No / Not applicable | Not reported. Feasibility study, interaction analyses were out of scope. |
| 12c | Explain how missing data were addressed | Yes | Methods: Multiple imputation (MICE) |
| 12d | Explain handling of follow‑up or matching | Yes | Methods: Comparison group and follow‑up characteristics |
| 12e | Describe sensitivity analyses | Yes | Methods: Sensitivity analysis section |
| 13a | Report numbers at each study stage | Yes | Results: Veteran identification and matching |
| 13b | Reasons for non-participation | Yes | Methods: Study population; veteran identification strategy and matching |
| 13c | Use of flow diagram | No / Not applicable | Not applicable |
| 14a | Participant characteristics | Yes | Results: Table 1 |
| 14b | Missing data for variables | Yes | Tables 1 and 2 (missing data reported) |
| 14c | Summarise follow‑up time | Yes | Table 1: Follow-up time |
| 15 | Report outcome events or summary measures | Yes | Table 3: Health outcomes |
| 16a | Unadjusted and adjusted estimates with CI | Yes | Table 4: Poisson regression results |
| 16b | Report category boundaries for variables | Yes | Methods: BMI and blood pressure definitions |
| 16c | Translate relative risk to absolute risk (if relevant) | No / Not applicable | Not reported |
| 17 | Report additional analyses | Yes | Results: Sensitivity analyses |
| 18 | Summarise key results | Yes | Discussion: First paragraph |
| 19 | Discuss study limitations | Yes | Discussion: Strengths and limitations |
| 20 | Provide cautious interpretation | Yes | Discussion: Interpretation and literature comparison |
| 21 | Discuss generalisability | Yes | Discussion: Implications and generalisability |
| 22 | Provide funding information | Yes | Declarations: Funding |

**Sent:**

10 patient IDs (5 veterans and 5 non-veterans) to 50 practices (50% had veteran friendly accreditation)

**Received:**

95 patients from 10 practices (70% veteran friendly)

34 (15 veterans and 19 non-veterans from 9 practices) did not have access to patient data (patient had left practice or died)

**Valid responses:**

61 patients

**Figure S1.** Flowchart of CPRD data sent to PROVE and GP data received

| **Table S3.** CPRD Aurum (veteran and non-veteran) variables descriptives for sample with complete data (n=118,674) | | | | |
| --- | --- | --- | --- | --- |
|  | **Non-veterans**  44,198 | | **Veterans**  74,476 | |
|  | N | % / Median [IQR] / Mean (SD) | N | % / Median [IQR] / Mean (SD) |
| **Gender** |  |  |  |  |
| Men | 31,930 | 72.24 | 58,901 | 79.09 |
| Women | 12,268 | 27.76 | 15,575 | 20.91 |
| **Age at index (years)** |  | 55.63 (19.85) |  | 54.80 (19.92) |
| 18-25 | 3,402 | 7.70 | 6,148 | 8.26 |
| 26-35 | 5,268 | 11.92 | 9,356 | 12.56 |
| 36-45 | 5,792 | 13.10 | 10,362 | 13.91 |
| 46-55 | 7,379 | 16.70 | 12,373 | 16.61 |
| 56-65 | 7,291 | 16.50 | 12,092 | 16.24 |
| 66-75 | 6,094 | 13.79 | 9,929 | 13.33 |
| 76-85 | 6,366 | 14.40 | 9,887 | 13.28 |
| 86-95 | 2,498 | 5.65 | 4,134 | 5.55 |
| 96+ | 108 | 0.24 | 195 | 0.26 |
| **Ethnicity** |  |  |  |  |
| Asian | 1,742 | 3.94 | 1,993 | 2.68 |
| Black | 603 | 1.36 | 1,097 | 1.47 |
| Mixed | 276 | 0.62 | 534 | 0.72 |
| Other | 3,578 | 8.10 | 5,483 | 7.36 |
| White | 37,999 | 85.97 | 65,369 | 87.77 |
| **Year of index** |  |  |  |  |
| 1987-1990 | 142 | 0.32 | 515 | 0.69 |
| 1991-2000 | 838 | 1.90 | 2,651 | 3.56 |
| 2001-2010 | 5,876 | 13.29 | 8,499 | 11.41 |
| 2011-2020 | 29,836 | 67.51 | 45,051 | 60.49 |
| 2021-2024 | 7,506 | 16.98 | 17,760 | 23.85 |
| **Follow-up time (years)*** |  | 5.40 [3.06-7.87] |  | 3.69 [1.72-6.39] |
| **Primary care consultations** |  | 10.32 [6.80-15.74] |  | 11.65 [7.20-18.25] |
| **Mortality** |  |  |  |  |
| Deceased | 4,209 | 9.52 | 7,218 | 9.69 |
| **Region** |  |  |  |  |
| North East | 5,604 | 12.68 | 7,694 | 10.33 |
| North West | 13,199 | 29.86 | 20,225 | 27.16 |
| Yorkshire and the Humber | 1,964 | 4.44 | 3,047 | 4.09 |
| East Midlands | 1,210 | 2.74 | 2,066 | 2.77 |
| West Midlands | 5,334 | 12.07 | 8,787 | 11.80 |
| East of England | 830 | 1.88 | 1,635 | 2.20 |
| London | 1,258 | 2.85 | 2,456 | 3.30 |
| South East | 11,429 | 25.86 | 21,564 | 28.95 |
| South West | 3,338 | 7.55 | 6,948 | 9.33 |
| Northern Ireland | 32 | 0.07 | 54 | 0.07 |
| SD: Standard deviation; IQR: Interquartile Range  Proportions calculated for patients with complete data on variables  *Start date calculated as maximum value between registration date, index date, and protocol start date (01 of January 1987) and end date calculated as minimum value between registration end date, last CPRD collection date for the practice, date of death, or protocol end date (30 of September 2024) | | | | |

| **Table S4.** Poisson regression for the association between veteran status and health conditions using complete case sample (n=118,674) | | | | | | | | | |
| --- | --- | --- | --- | --- | --- | --- | --- | --- | --- |
|  | **Model 1** | | | **Model 2** | | | **Model 3** | | |
|  | PR | 95% CI | | aPR | 95% CI | | aPR | 95% CI | |
| **Alcohol use disorder** | 1.16 | 1.06 | 1.28 | 1.16 | 1.05 | 1.27 | 1.14 | 1.03 | 1.25 |
| **Alzheimer’s disease** | 1.64 | 1.49 | 1.81 | 1.62 | 1.47 | 1.78 | 1.47 | 1.34 | 1.62 |
| **Anxiety** | 0.95 | 0.92 | 0.99 | 0.95 | 0.92 | 0.99 | 0.95 | 0.91 | 0.98 |
| **Breast cancer** | 0.96 | 0.83 | 1.11 | 0.96 | 0.83 | 1.11 | 0.93 | 0.80 | 1.07 |
| **Coronary heart disease** | 1.01 | 0.97 | 1.05 | 1.01 | 0.97 | 1.05 | 0.97 | 0.93 | 1.01 |
| **Chronic obstructive pulmonary disease** | 1.08 | 1.04 | 1.13 | 1.07 | 1.03 | 1.11 | 1.02 | 0.98 | 1.06 |
| **Depression** | 1.12 | 1.09 | 1.16 | 1.12 | 1.08 | 1.16 | 1.10 | 1.07 | 1.14 |
| **Hearing loss** | 1.23 | 1.18 | 1.28 | 1.22 | 1.17 | 1.27 | 1.18 | 1.14 | 1.23 |
| **Lower back pain** | 0.99 | 0.96 | 1.02 | 0 .99 | 0.96 | 1.02 | 0.98 | 0.95 | 1.00 |
| **Myocardial infarction** | 0.93 | 0.86 | 1.00 | 0.93 | 0.86 | 1.00 | 0.90 | 0.84 | 0.98 |
| **Osteoarthritis** | 1.08 | 1.04 | 1.11 | 1.07 | 1.04 | 1.11 | 1.04 | 1.01 | 1.08 |
| **Prostate cancer^*^** | 1.03 | 0.96 | 1.12 | 1.02 | 0.94 | 1.10 | 0.97 | 0.89 | 1.05 |
| **Post-traumatic stress disorder** | 8.61 | 7.29 | 10.17 | 8.55 | 7.24 | 10.09 | 8.27 | 7.00 | 9.76 |
| Men patients only (n=90,831)  PR: Prevalence Ratios; aPR: Adjusted Prevalence Ratios; CI: Confidence Interval  Model 1: gender, age, index date and GP practice; Model 2: Model 1 + ethnicity; Model 3: Model 2 + BMI and smoking | | | | | | | | | |

| **Table S5.** Poisson regression for the association between veteran status and health conditions setting missing in health risk factors to “healthy” categories (N=367,057) | | | | | | | | | |
| --- | --- | --- | --- | --- | --- | --- | --- | --- | --- |
|  | **Model 1** | | | **Model 2** | | | **Model 3** | | |
|  | PR | 95% CI | | aPR | 95% CI | | aPR | 95% CI | |
| **Alcohol use disorder** | 2.32 | 2.18 | 2.48 | 2.25 | 2.11 | 2.41 | 1.66 | 1.55 | 1.79 |
| **Alzheimer’s disease** | 2.83 | 2.65 | 3.01 | 2.61 | 2.45 | 2.79 | 2.40 | 2.23 | 2.58 |
| **Anxiety** | 1.53 | 1.50 | 1.57 | 1.44 | 1.40 | 1.47 | 1.20 | 1.17 | 1.23 |
| **Breast cancer** | 1.47 | 1.31 | 1.65 | 1.46 | 1.29 | 1.64 | 1.14 | 1.01 | 1.30 |
| **Coronary heart disease** | 1.95 | 1.89 | 2.02 | 1.87 | 1.81 | 1.94 | 1.22 | 1.17 | 1.26 |
| **Chronic obstructive pulmonary disease** | 2.06 | 1.99 | 2.13 | 1.91 | 1.85 | 1.98 | 1.13 | 1.10 | 1.17 |
| **Depression** | 1.89 | 1.85 | 1.93 | 1.79 | 1.75 | 1.83 | 1.46 | 1.42 | 1.49 |
| **Hearing loss** | 2.20 | 2.13 | 2.27 | 2.01 | 1.94 | 2.07 | 1.56 | 1.51 | 1.61 |
| **Lower back pain** | 1.80 | 1.76 | 1.84 | 1.70 | 1.66 | 1.74 | 1.28 | 1.25 | 1.31 |
| **Myocardial infarction** | 1.81 | 1.71 | 1.91 | 1.75 | 1.65 | 1.85 | 1.14 | 1.07 | 1.21 |
| **Osteoarthritis** | 2.13 | 2.08 | 2.18 | 1.97 | 1.92 | 2.02 | 1.37 | 1.33 | 1.40 |
| **Prostate cancer^*^** | 2.00 | 1.89 | 2.12 | 1.85 | 1.74 | 1.96 | 1.34 | 1.26 | 1.43 |
| **Post-traumatic stress disorder** | 17.08 | 15.47 | 18.85 | 16.09 | 14.58 | 17.77 | 14.04 | 12.68 | 15.55 |
| *Men patients only (n=294,876)  PR: Prevalence Ratios; aPR:Adjusted Prevalence Ratios; CI: Confidence Interval  Model 1: Unadjusted; Model 2: Model 1 + ethnicity; Model 3: Model 2 + BMI and smoking | | | | | | | | | |

| **Table S6**. CPRD Aurum sample (veteran and non-veteran) descriptives for patients identified/registered in 2014 or after (n=286,359) | | | | | |
| --- | --- | --- | --- | --- | --- |
|  |  | **Non-veterans** 190,797 | | **Veterans** 95,562 | |
|  |  | **N** | **% or Mean (SD) [IQR]** | **N** | **% or Mean (SD) [IQR]** |
| **Gender** | |  |  |  |  |
|  | Men | 159,897 | 83.80 | 80,092 | 83.81 |
|  | Women | 30,900 | 16.20 | 15,470 | 16.19 |
| **Age at index** | |  | 55.01 (20.78) |  | 55.01 (20.78) |
|  | 18-25 | 15,530 | 8.14 | 7,782 | 8.14 |
|  | 26-35 | 28,035 | 14.69 | 14,046 | 14.70 |
|  | 36-45 | 25,026 | 13.12 | 12,541 | 13.12 |
|  | 46-55 | 29,837 | 15.64 | 14,945 | 15.64 |
|  | 56-65 | 28,798 | 15.09 | 14,423 | 15.09 |
|  | 66-75 | 22,873 | 11.99 | 11,453 | 11.98 |
|  | 76-85 | 25,786 | 13.51 | 12,909 | 13.51 |
|  | 86-95 | 13,880 | 7.27 | 6,947 | 7.27 |
|  | 96+ | 1,032 | 0.54 | 516 | 0.54 |
| **Ethnicity** | |  |  |  |  |
|  | Asian | 9,205 | 5.43 | 2,131 | 2.34 |
|  | Black | 3,450 | 2.03 | 1,369 | 1.50 |
|  | Mixed | 2,489 | 1.47 | 759 | 0.83 |
|  | Other | 15,015 | 8.86 | 5,634 | 6.17 |
|  | White | 139,399 | 82.21 | 81,355 | 89.16 |
|  | *Missing* | *21,239* | *11.13* | *4,314* | *4.51* |
| **Year of index** | |  |  |  |  |
|  | 2014-2020 | 124,288 | 65.14 | 62,215 | 65.10 |
|  | 2021-2024 | 66,509 | 34.86 | 33,347 | 34.90 |
| **Follow-up time (years)*** | |  | 2.73 [1.11-5.09] |  | 2.49 [1.11-4.70] |
| **Primary care consultations** | |  | 6.66 [4.22-11.00] |  | 10.11 [6.00-16.46] |
| **Mortality** | |  |  |  |  |
|  | Deceased | 9,112 | 4.78 | 8,912 | 9.33 |
| **Year of death** | |  |  |  |  |
|  | 2014-2020 | 5,046 | 55.38 | 3,503 | 39.31 |
|  | 2021-2024 | 4,066 | 44.62 | 5,409 | 60.69 |
| **Region** | |  |  |  |  |
|  | North East | 18,349 | 9.62 | 9,183 | 9.61 |
|  | North West | 52,999 | 27.78 | 26,591 | 27.83 |
|  | Yorkshire and the Humber | 6,391 | 3.35 | 3,197 | 3.35 |
|  | East Midlands | 5,364 | 2.81 | 2,685 | 2.81 |
|  | West Midlands | 20,923 | 10.97 | 10,474 | 10.96 |
|  | East of England | 4,169 | 2.19 | 2,087 | 2.18 |
|  | London | 5,634 | 2.95 | 2,818 | 2.95 |
|  | South East | 57,605 | 30.19 | 28,837 | 30.18 |
|  | South West | 19,193 | 10.06 | 9,605 | 10.05 |
|  | Northern Ireland | 170 | 0.09 | 85 | 0.09 |
| Category percentages are based on complete cases; missing percentages are based on the full sample.  SD: Standard deviation; IQR: Interquartile Range  *Start date calculated as maximum value between registration date, index date, and protocol start date (01 of January 1987) and end date calculated as minimum value between registration end date, last CPRD collection date for the practice, date of death, or protocol end date (30 September 2024). | | | | | |

| **Table S7.** CPRD Aurum sample (veteran and non-veteran) health risk factors for patients identified/registered in 2014 or after (n=286,359) | | | | | | | | | | | | | | | |
| --- | --- | --- | --- | --- | --- | --- | --- | --- | --- | --- | --- | --- | --- | --- | --- |
|  |  | **Non-veterans 190,797** | | | | | | | **Veterans 95,562** | | | | | | |
|  |  | **N** | **%** | **95% CI*** | | **%** | **95% CI**† | | **N** | **%** | **95% CI*** | | **%** | **95% CI**† | |
| **Smoker** |  |  |  |  |  |  |  |  |  |  |  |  |  |  |  |
|  | Never | 39,097 | 51.38 | 51.02 | 51.73 | 53.97 | 53.65 | 54.28 | 33,634 | 45.54 | 45.54 | 45.18 | 46.34 | 45.97 | 46.72 |
|  | Ex-smoker | 22,324 | 29.34 | 29.01 | 29.66 | 27.72 | 27.39 | 28.05 | 26,834 | 36.33 | 35.98 | 36.68 | 35.55 | 35.16 | 35.93 |
|  | Smoker | 14,675 | 19.28 | 19.01 | 19.57 | 18.31 | 18.00 | 18.62 | 13,396 | 18.14 | 17.86 | 18.42 | 18.11 | 17.86 | 18.36 |
|  | *Missing* | *114,701* | *60.12* | *-* | *-* | *-* | *-* | *-* | *21,698* | *22.71* | *-* | *-* | *-* | *-* | *-* |
| **Blood pressure** |  |  |  |  |  |  |  |  |  |  |  |  |  |  |  |
|  | Normal | 15,957 | 19.29 | 19.02 | 19.56 | 19.66 | 19.30 | 20.02 | 11,773 | 17.31 | 17.03 | 17.60 | 18.20 | 17.91 | 18.48 |
|  | Pre-hypertension | 39,252 | 47.45 | 47.11 | 47.79 | 48.09 | 47.74 | 48.43 | 33,177 | 48.78 | 48.40 | 49.15 | 48.84 | 48.47 | 49.21 |
|  | Hypertension | 27,511 | 33.26 | 32.94 | 33.58 | 32.25 | 31.89 | 32.62 | 23,065 | 33.91 | 33.56 | 34.27 | 32.97 | 32.60 | 33.33 |
|  | *Missing* | *108,077* | *56.65* | *-* | *-* | *-* | *-* | *-* | *27,547* | *28.83* | *-* | *-* | *-* | *-* | *-* |
| **BMI** |  |  |  |  |  |  |  |  |  |  |  |  |  |  |  |
|  | Underweight | 3,325 | 5.24 | 5.07 | 5.41 | 4.27 | 4.11 | 4.44 | 861 | 1.37 | 1.28 | 1.46 | 2.00 | 1.87 | 2.13 |
|  | Normal weight | 17,235 | 27.14 | 26.80 | 27.49 | 29.09 | 28.78 | 29.41 | 17,009 | 27.05 | 26.70 | 27.40 | 27.05 | 26.74 | 27.37 |
|  | Overweight | 22,381 | 35.25 | 34.88 | 35.62 | 36.62 | 36.24 | 36.99 | 24,618 | 39.15 | 38.77 | 39.53 | 38.47 | 38.11 | 38.83 |
|  | Obesity class I | 13,097 | 20.63 | 20.31 | 20.94 | 19.75 | 19.36 | 20.14 | 13,770 | 21.90 | 21.58 | 22.22 | 21.70 | 21.36 | 22.03 |
|  | ≥Obesity class II | 7,461 | 11.75 | 11.50 | 12.00 | 10.27 | 10.06 | 10.48 | 6,628 | 10.54 | 10.30 | 10.78 | 10.78 | 10.52 | 11.05 |
|  | *Missing* | *127,298* | *66.72* | *-* | *-* | *-* | *-* | *-* | *32,676* | *34.19* | *-* | *-* | *-* | *-* | *-* |
| Category percentages are based on complete cases; missing percentages are based on the full sample.  *Proportions calculated for patients with complete data on variables  †Pooled proportions obtained using multiple imputed datasets | | | | | | | | | | | | | | | |

| **Table S8.** CPRD Aurum sample (veteran and non-veteran) health profile for patients identified/registered in 2014 or after (n=286,359) | | | | | | | | | |
| --- | --- | --- | --- | --- | --- | --- | --- | --- | --- |
|  | | **Non-veterans** | | | | **Veterans** | | | |
|  | | **N** | **%** | **95% CI** | | **N** | **%** | **95% CI** | |
| **Alcohol use disorder** | | 1,174 | 0.62 | 0.58 | 0.65 | 1,284 | 1.34 | 1.27 | 1.42 |
| **Alzheimer’s disease** | | 1,119 | 0.59 | 0.55 | 0.62 | 1,827 | 1.91 | 1.83 | 2.00 |
| **Anxiety** | | 10,496 | 5.50 | 5.40 | 5.60 | 7,189 | 7.52 | 7.36 | 7.69 |
| **Breast cancer** | | 337 | 0.18 | 0.16 | 0.20 | 227 | 0.24 | 0.21 | 0.27 |
| **CHD** | | 4,259 | 2.23 | 2.17 | 2.30 | 4,795 | 5.02 | 4.88 | 5.16 |
| **COPD** | | 4,840 | 2.54 | 2.47 | 2.61 | 5,474 | 5.73 | 5.58 | 5.88 |
| **Depression** | | 9,928 | 5.20 | 5.10 | 5.30 | 8,622 | 9.02 | 8.84 | 9.21 |
| **Hearing loss** | | 5,163 | 2.71 | 2.63 | 2.78 | 6,210 | 6.50 | 6.34 | 6.66 |
| **Lower Back Pain** | | 10,471 | 5.49 | 5.39 | 5.59 | 9,068 | 9.49 | 9.30 | 9.68 |
| **Myocardial Infarction** | | 1,528 | 0.80 | 0.76 | 0.84 | 1,558 | 1.63 | 1.55 | 1.71 |
| **Osteoarthritis** | | 7,391 | 3.87 | 3.79 | 3.96 | 8,095 | 8.47 | 8.30 | 8.65 |
| **Prostate cancer*** | | 1,741 | 1.09 | 1.04 | 1.14 | 1,924 | 2.40 | 2.30 | 2.51 |
| **PTSD** | | 339 | 0.18 | 0.16 | 0.20 | 2,935 | 3.07 | 2.96 | 3.18 |
| **Multimorbidity** | |  |  |  |  |  |  |  |  |
|  | 1 condition | 24,936 | 63.26 | 62.78 | 63.73 | 21,582 | 58.49 | 57.98 | 58.99 |
|  | 2 conditions | 10,728 | 27.21 | 26.78 | 27.66 | 10,168 | 27.56 | 27.10 | 28.01 |
|  | 3 conditions | 2,890 | 7.33 | 7.08 | 7.59 | 3,732 | 10.11 | 9.81 | 10.43 |
|  | 4 or more conditions | 867 | 2.20 | 2.06 | 2.35 | 1,418 | 3.84 | 3.65 | 4.04 |
| *Men patients only (n=239,989)  CHD: Coronary Heart Disease; COPD: Chronic obstructive pulmonary disease; PTSD: Post-traumatic stress disorder; BMI: Body mass index. | | | | | | | | | |

| **Table S9.** Poisson regression for the association between veteran status and health conditions for patients identified/registered in 2014 or after (n=286,359) | | | | | | | | | |
| --- | --- | --- | --- | --- | --- | --- | --- | --- | --- |
|  | **Model 1** | | | **Model 2** | | | **Model 3** | | |
|  | PR | 95% CI | | aPR | 95% CI | | aPR | 95% CI | |
| **Alcohol use disorder** | 2.18 | 2.01 | 2.36 | 2.10 | 1.94 | 2.27 | 2.08 | 1.92 | 2.26 |
| **Alzheimer’s disease** | 3.25 | 3.02 | 3.51 | 3.10 | 2.88 | 3.34 | 3.09 | 2.86 | 3.33 |
| **Anxiety** | 1.36 | 1.32 | 1.40 | 1.33 | 1.29 | 1.37 | 1.33 | 1.29 | 1.37 |
| **Breast cancer** | 1.34 | 1.13 | 1.59 | 1.31 | 1.11 | 1.56 | 1.31 | 1.10 | 1.55 |
| **Coronary heart disease** | 2.24 | 2.15 | 2.34 | 2.16 | 2.07 | 2.25 | 2.01 | 1.92 | 2.09 |
| **Chronic obstructive pulmonary disease** | 2.25 | 2.17 | 2.34 | 2.15 | 2.07 | 2.24 | 1.96 | 1.89 | 2.04 |
| **Depression** | 1.73 | 1.68 | 1.78 | 1.68 | 1.64 | 1.73 | 1.67 | 1.62 | 1.72 |
| **Hearing loss** | 2.40 | 2.31 | 2.48 | 2.31 | 2.22 | 2.39 | 2.24 | 2.16 | 2.32 |
| **Lower back pain** | 1.72 | 1.68 | 1.77 | 1.70 | 1.65 | 1.75 | 1.65 | 1.60 | 1.69 |
| **Myocardial infarction** | 2.03 | 1.89 | 2.18 | 1.95 | 1.81 | 2.09 | 1.83 | 1.70 | 1.96 |
| **Osteoarthritis** | 2.18 | 2.12 | 2.25 | 2.10 | 2.04 | 2.17 | 1.99 | 1.93 | 2.06 |
| **Prostate cancer^*^** | 2.20 | 2.06 | 2.35 | 2.10 | 1.97 | 2.24 | 2.00 | 1.88 | 2.14 |
| **Post-traumatic stress disorder** | 17.28 | 15.45 | 19.33 | 17.05 | 15.24 | 19.07 | 16.93 | 15.13 | 18.94 |
| *Men patients only (n=239,989)  PR: Prevalence Ratios; aPR:Adjusted Prevalence Ratios; CI: Confidence Interval  Model 1: Gender, age, index date, and GP practice; Model 2: Model 1 + ethnicity; Model 3: Model 2 + BMI and smoking | | | | | | | | | |
